# Does size still matter? - Feasibility of posterior retroperitoneoscopic adrenalectomy for tumors >6cm

**DOI:** 10.1007/s00423-025-03769-7

**Published:** 2025-06-13

**Authors:** Joy Feka, Barbara Soliman, Melisa Arikan, Magdalena Sacher, Teresa Binter, Lindsay Hargitai, Christian Scheuba, Philipp Riss

**Affiliations:** Department of General Surgery, Division of Visceral Surgery, Medical University of Vienna, Waehringer Guertel 18-20, Vienna, 1090 Austria

**Keywords:** Adrenal tumor, Retroperitoneoscopic adrenalectomy, Large tumor size, RPA, Cut-off, Minimal-invasive surgery

## Abstract

**Purpose:**

Retroperitoneoscopic adrenalectomy (RPA) has proven to be safe and feasible with favorable postoperative courses. The role of RPA for tumor sizes larger than 6 cm is still controversial. The aim of the study was to evaluate the postoperative outcome for removal of larger adrenal tumors via the retroperitoneoscopic route.

**Methods:**

In this retrospective study, from 105 conducted RPA procedures, thirteen patients with adrenal tumor sizes larger than 6 cm received RPA in our hospital between January 2017 and December 2020. Clinicopathological factors, length of hospital stay, operative time and postoperative outcomes were included in this analysis.

**Results:**

From this patient cohort, six (46.15%) were female and seven (53.85%) were male with a mean age of 53.85 ± 7.89 years and a mean BMI of 28.64 ± 3.61 kg/m2, Cushing’s syndrome being the most common diagnosis (53.85%). Mean lesion size was 73.31 ± 10.39 mm, tumor size varied from 60 mm up to 92 mm. Two patients (15.38%) required conversion to open laparotomy due to uncontrollable bleeding or an unclear view on the basis of adhesions. Postoperative complications were noted for one patient (7.69%), who suffered from a small superficial wound infection. Neither capsule ruptures nor mortality were documented. The median hospital stay was 3 days.

**Conclusion:**

A re-evaluation of the arbitrarily placed cut-off should be discussed, since even with a slightly higher but nevertheless acceptable risk of conversion rate, RPA offers many advantages.

## Introduction

There are three different techniques to successfully perform an adrenalectomy: open via laparoscopic transperitoneal or retroperitoneoscopic access [1]. Minimally invasive surgery (MIS) has many advantages, also in the endocrine surgical field [2–4]. Retroperitoneoscopic adrenalectomy (RPA) has proven to be safe and feasible, and shows to be beneficial due to direct access to the adrenal gland, leaving the peritoneal cavity intact and offering an easier operative setting in obese patients [5–7]. According to current guidelines from the German Association of Endocrine Surgeons (CAEK), both techniques are safe and the surgeon may choose the preferred technique depending on expertise for tumor sizes ≤ 6 cm [8, 9]. If the adrenal mass exceeds this cut-off, an open approach should be chosen due to risk of malignancy [6, 10]. The risk of malignant origin increases exponentially from 5% in tumor sizes of 5–8 cm to > 60% in mass sizes above 8 cm [6, 8].

The objective of this retrospective study was to evaluate the safety and feasibility of performing adrenalectomy with tumor sizes larger than 6 cm in a retroperitoneoscopic approach.

## Methods

### Patient collective

This retrospective study included 13 consecutive patients who had received RPA at a tertiary referral care center from January 2017 to December 2020 for tumors larger than 6 cm. Clinicopathological factors, length of hospital stay, operative time and postoperative outcomes were included in this analysis.

The first intervention with RPA was performed at our department in 2017. In that year, 33 patients were operated with this new procedure, and 105 RPAs in total were carried out from 2017 until the end of 2020. Thirteen patients, who showed no clear signs of malignancy (no signs of invasion or tumor necrosis), were selected to be operated via the retroperitoneoscopic access route. Since the new technique was established, most patients received RPA.

Preoperative assessment of tumor malignancy was conducted using a combination of imaging modalities, clinical evaluation, and biochemical testing. All patients underwent contrast-enhanced CT scans or MRI of the abdomen to evaluate tumor size, morphology, and specific features suggestive of malignancy, such as irregular borders, heterogeneity, or the presence of enlarged lymph nodes and visible tumor necrosis. FDG-PET scans were not performed in all patients. In cases with a high risk of malignancy, patients underwent laparoscopic transabdominal dissection with planned conversion to prevent capsule rupture.

For patients with hormonally active tumors, a specific perioperative medical protocol was followed to optimize surgical outcomes and minimize potential complications. All patients with suspected hormonally active adrenal tumors underwent preoperative biochemical testing, including measurement of catecholamines and metanephrines for suspected pheochromocytoma, as well as serum aldosterone, renin, cortisol, and plasma ACTH levels to assess for primary aldosteronism or Cushing’s syndrome.

In the case of pheochromocytomas, alpha-blockade was initiated preoperatively to manage hypertension and prevent intraoperative hypertensive crises. This was started 7–10 days before surgery and adjusted according to blood pressure and heart rate. This procedure is currently under evaluation, as recent studies have questioned the necessity of routine perioperative α-receptor blockade in phaeochromocytoma surgery, showing only minimal differences in intraoperative blood pressure and no major differences in complication rates between patients with and without blockade [11]. In patients with primary aldosteronism, spironolactone was administered to correct electrolyte imbalances and control blood pressure. For patients with Cushing’s syndrome, hydrocortisone was administered to prevent adrenal insufficiency following resection.

Due to the small number of patients, this is a preliminary analysis.

### Surgery

The patients were set in a prone jack-knife position for the RPA procedures. This can be particularly useful when bilateral adrenalectomy is indicated. Omitting intraoperative patient repositioning, which is necessary during laparoscopic transperitoneal adrenalectomy (LTA), the operative time was immensely reduced. Trocars were placed as follows: the first 12 mm trocar was placed below the lowest tip of the 12 th rib in the medioclavicular line. The capnoperitoneum was then applied: CO2 insufflation with a pressure peak of at least 20 mmHg was set, which is higher when compared to LTA. This is beneficial for a clearer view during dissection, since small vessels are compressed due to the high pressure. A 12 mm and a 5 mm trocar were then both placed below the 12 th rib on the right and left side of the already placed trocar. A 30° camera was used. The gland and tumor were retrieved in an endobag.

The operating time was defined as skin-to-skin time.

Information on tumor size had been collected from histological findings. When bilateral adrenalectomies were conducted, both tumor sizes were used for calculations.

### Postoperative data

Hospital stay was defined as the time between the days of admission and discharge.

Routine check-ups were done two weeks after discharge at the outpatient clinic of the department of general surgery and 6 weeks postoperatively at the department of endocrinology.

### Statistical analysis

Continuous variables were presented as the mean values ± standard deviation. Patient and tumor characteristics, including age, body mass index and tumor size, were collected. Clinical outcomes, such as operative time, length of stay and postoperative complications, were also noted. Due to the small patient collective, only descriptive statistics could be conducted. All statistical analyses were performed using SPSS Statistics ver. 22.0 (IBM, Armonk, NY, USA).

### Ethical statement

The study was conducted in accordance with the declaration of Helsinki (as revised in 2013). The study was approved by the institutional ethical review board (EK nr. 1170/2023).

## Results

During the time period between January 2017 and December 2020, 105 patients were submitted to RPA. Out of these patients, thirteen were found to have tumor sizes exceeding 6 cm (Table 1).

**Table 1:** Total study cohort

Pat. #	Sex	Age (years)	BMI (kg/m²)	OP date	OP time (min)	Side	Conversion	Complications	Diagnosis	Histopathology	Tumor size (mm)
1	Male	50	28.1	2017	235	Bilateral	Bleeding	No	Cushing	Adrenal cortical adenoma	80/70
2	Female	44	27.2	2018	95	Bilateral	No	No	Cushing	Adrenal cortical adenoma	80/63
3	Male	69	30.4	2018	80	Left	No	Wound infection	Pheochromocytoma	Pheochromocytoma	60
4	Male	47	28.9	2018	50	Left	No	No	Pheochromocytoma	Pheochromocytoma	92
5	Male	65	22.9	2018	95	Left	No	No	Conn	Adrenal cortical adenoma	70
6	Male	77	23.9	2018	100	Bilateral	No	No	Incidentalioma	Adrenal lymphoma	60/42
7	Male	56	27.8	2018	50	Right	Adhesions	No	Incidentalioma	Adrenal cortical adenoma	90
8	Male	63	25.3	2019	145	Left	No	No	Metastasis	Metastasis to adrenal gland	76
9	Female	63	32.7	2020	50	Left	No	No	Cushing	Adrenal cortical adenoma	60
10	Female	46	39.0	2020	90	Bilateral	No	No	Cushing	Adrenal cortical hyperplasia	70/35
11	Female	49	40.5	2020	20	Bilateral	No	No	Cushing	Adrenal cortical hyperplasia	80/57
12	Female	30	19.4	2020	35	Right	No	No	Cushing	Adrenal cortical adenoma	70
13	Female	41	26.2	2020	40	Right	No	No	Cushing	Adrenal cortical adenoma	65

### Diagnoses and histological findings

Six patients (46.15%) in our patient cohort were female and seven (53.85%) male. The mean age was 53.85 ± 7.89 years and mean BMI was 28.64 ± 3.61 kg/m2. The most common preoperative diagnoses were Cushing’s syndrome (*n* = 7; 53.85%), pheochromocytoma (*n* = 2; 15.38%), incidentalioma/adenoma (*n* = 2;15.38%), Conn’s syndrome (*n* = 1; 7.69%) and metastasis to the adrenal gland (*n* = 1; 7.69%). The mean tumor size was 73.31 ± 10.39 mm, ranging from 60 mm up to 92 mm. Five tumors (38.46%) were located on the left side, three (23.08%) on the right, and 5 patients (38.46%) had bilateral adrenal tumors.

Histopathological findings showed that the majority of patients (*n* = 7) had adrenal cortical adenoma (53.85%), followed by two adrenal cortical hyperplasia (15.38%), two pheochromocytoma (15.38%), one adrenal lymphoma (7.69%) and one metastasis to the adrenal gland from cancer of unknown primary (CUP) (7.69%).

### Complications

Eleven patients (84.62%) received an uncomplicated procedure, whereas two patients (15.38%) needed conversion to open laparotomy: one patient (7.69%) due to uncontrollable bleeding and another (7.69%) due to unclear view on the basis of adhesions. The mean operation time was 83.46 ± 34.44 min including all patients, but when excluding the two conversion cases, the mean operative time was 72.73 ± 24.79 min. In comparison with all patients undergoing unilateral RPA at our department, the mean operative time was 61.68 ± 8.22 min. The longer operative time can be deduced as a consequence of larger tumor size, hence leading to more difficult operating conditions (Table 2). Estimated intraoperative blood loss was minimal in the majority of cases and was not systematically recorded for every procedure due to the retrospective nature of the study. Systematic postoperative pain scores (e.g., using a visual analog scale) were not routinely collected during the study period. In general, all patients received standardized pain management protocols, which included non-opioid analgesics (such as intravenous or oral metamizol and/or NSAIDs) as first-line therapy. Opioids were reserved for breakthrough pain if necessary, but were only infrequently required in cases where conversion was necessary.

**Table 2:** Postoperative outcome

	Total	
*Number of cases*	13	
*Sex (female: male)*	6: 7	46.2%: 53.8%
*Mean age (years)*	53.85 ± 7.88	
*Mean BMI (kg/*m^2^*)*	28.64 ± 3.61	
*Operation side*		
*(right: left)*	3:5	23.1%: 38.5%
*(bilateral)*	5	38.5%
*Mean tumor size (mm)*	73.31 ± 10.39	60–92 range
*Median operating time (min)*	80	20–235
*Median hospital stay (days)*	3	1–14
*Conversion*	2	15.4%
*Postoperative complications*	1	7.7%

Postoperative complications were noted for one patient (7.69%), who suffered from a small superficial wound infection. No capsule ruptures nor mortality were documented. The median hospital stay was 3 days. The prolonged hospital stay of 14 days occurred in a patient who experienced significant postoperative blood loss, which required extended monitoring and supportive care. Additionally, this patient underwent conversion from RPA to an open procedure, which was associated with increased postoperative pain and a slower recovery, contributing to the longer hospitalization period.

### Follow up

All patients underwent clinical evaluations at 2 weeks and 6 weeks postoperatively, followed by annual follow-up visits thereafter. At the 30-day postoperative assessment, no complications of Clavien-Dindo grade II or higher were observed, and the early postoperative course was uneventful across the cohort. In patients with hormone-secreting tumors, clinical symptoms resolved entirely following adrenalectomy. Biochemical follow-up confirmed normalization of hormonal levels in all cases, indicating successful symptomatic and biochemical control.

## Discussion

The number of newly diagnosed adrenal tumors has risen due to an increased number of completed abdominal imaging studies. When an adrenal tumor is detected, a thorough evaluation is necessary to differentiate between hormone active and inactive tumors as well as benign and malignant origins [12]. If surgical resection is indicated, there are three different techniques to choose from: open adrenalectomy, LTA or RPA [1]. Apart from the open approach, LTA was a new accomplishment in MIS in the field of surgical endocrinology, first described by Gagner and Higashihara in 1992 [2–4]. Shortly thereafter, Brunt et al. started to experiment with retroperitoneoscopic access on animal models as another possible MIS procedure [13]. The advantages of MIS can generally also be found in adrenalectomies: better quality of life, less intraoperative blood loss and postoperative pain, quicker return to everyday life, shorter hospital stay and reduced mortality [5–7].

The RPA procedure was revised by Walz et al. in 1995, who performed RPA in a prone jack-knife position [14]. Walz et al. described many advantages of RPA, including shorter operative time, hospital stay and return to everyday life [1, 15, 16]. Intraoperatively, the peritoneal sack need not be opened, hence no mobilization of abdominal organs is necessary. Especially in previously operated or obese patients, this procedure can be performed more easily and risk-free [17]. Nevertheless, the RPA technique does not receive high recognition due to more complicated anatomy and less expertise in this particular field [18, 19]. According to Walz, at least 30 cases are to be operated in order to for high-volume surgeons to gain the required experience for RPA, which is a high number only a few centers are able to fulfill [16].

Both techniques have proven to be safe and feasible, and due to their equality, both have been included in current guidelines [6, 20–22]. When comparing LTA with RPA, a slight superiority for RPA is noted, particularly in male or obese patients, as well as those with bilateral tumors [1].

However, the gold standard for adrenalectomies remains to be LTA, which can be attributed to the absence of sufficient experience, limited case numbers, and unfamiliarity with anatomical landmarks in this new access route.

Most surgeons feel safer in the transperitoneal laparoscopic setting, because the lack of anatomical landmarks in the retroperitoneum can be viewed as difficult, hence guidance by experienced surgeons at the beginning of the learning curve is crucial [23].

### Current guidelines

As one of the first recommendations in current guidelines of the CAEK, it is stated that the surgeon may choose the preferred MIS technique depending on expertise in tumor sizes ≤ 6 cm [8]. If the adrenal mass exceeds this cut-off, an open approach should be chosen due to risk of malignancy [6]. The risk of malignant origin exponentially increases from 5% in tumor sizes of 5–8 cm to > 60% in mass sizes above 8 cm [8]. But size alone is not an absolute criterion for a malignant origin [8, 22, 24, 25]. The American Association of Endocrine Surgeons guidelines do not specify tumor size, as this decision should be made by each surgeon according to experience and skill, while the British guidelines do not give any statement at all [25].

In 2021, a systematic review and meta-analysis by Meng et al. included nine studies with a total of 800 patients, where operative time, blood loss, postoperative pain, length of hospital stay were in favor to the RPA [24]. Here, tumors with larger than 8 cm were excluded from RPA treatment [24].

Our study shows that adrenalectomies for large tumors can be successfully performed via the retroperitoneal access. The thirteen patients, who had a bigger tumor size than 6 cm with no signs of malignancy, were operated by one of three experienced endocrine surgeons at our department. This new technique was introduced in 2017, initially in the presence of Prof. Martin Walz from Essen. In total, 105 patients were operated from January 2017 until the end of December 2020 [26]. This technique has shown to be advantageous for many reasons, also in our patient cohort. The conversion rate for all patients receiving RPA was approx. 3.81% [26]. Due to larger tumor sizes in our study, a more difficult surgical setting, the conversion rate was 15.38%. This higher number can be explained by the lack of experience at the beginning of the establishment for RPA in terms of larger tumor sizes. In 2019 and 2020, no conversions were necessary [26].

Although the data presently suggest that the clear cut-off of 6 cm should be intensively discussed, the need for more studies is crucial to further evaluate the definite superiority of RPA in comparison to LTA.

### Limitations

This study has several limitations that should be acknowledged. First, its retrospective design inherently carries a risk of selection bias. Second, the small sample size of only 13 patients limits the generalizability of the findings and precludes robust statistical analysis beyond descriptive reporting. Although definitive conclusions cannot be drawn, the study offers valuable clinical insights into a rare and specific patient population. Future prospective, multi-center studies with larger patient cohorts are needed to validate and expand upon these preliminary observations.

## Conclusion

Retroperitoneoscopic adrenalectomy has proven to be safe and feasible, while offering advantages in operative time, faster recovery time and an acceptable rate of complications, when performed by experienced endocrine surgeons. In contrast to current guidelines, this study indicates that retroperitoneoscopic adrenalectomy (RPA) is a feasible and safe technique for adrenal tumors larger than 6 cm. Although conversion rates were slightly higher in larger tumors, they remained within an acceptable range, and postoperative outcomes were favorable. Future prospective studies with larger patient cohorts are needed to confirm these findings and further define the role of RPA in adrenal surgery.

## Data Availability

No datasets were generated or analysed during the current study.

## References

[1] Walz MK (2022) [Minimally invasive techniques in adrenal gland surgery]. Chirurgie (Heidelb) 93(9):850–855 35927340 10.1007/s00104-022-01682-z

[2] Zacharias M, Haese A, Jurczok A, Stolzenburg JU, Fornara P (2006) Transperitoneal laparoscopic adrenalectomy: outline of the preoperative management, surgical approach, and outcome. Eur Urol 49(3):448–459 16481096 10.1016/j.eururo.2006.01.014

[3] Gagner M, Lacroix A, Bolte E (1992) Laparoscopic adrenalectomy in cushing’s syndrome and pheochromocytoma. N Engl J Med 327(14):1033 1387700 10.1056/NEJM199210013271417

[4] Higashihara E, Tanaka Y, Horie S, Aruga S, Nutahara K, Homma Y et al (1992) [A case report of laparoscopic adrenalectomy]. Nihon Hinyokika Gakkai Zasshi 83(7):1130–1133 1387181 10.5980/jpnjurol1989.83.1130

[5] Brunt LM, Doherty GM, Norton JA, Soper NJ, Quasebarth MA, Moley JF (1996) Laparoscopic adrenalectomy compared to open adrenalectomy for benign adrenal neoplasms. J Am Coll Surg 183(1):1–10 8673301

[6] Lee CR, Walz MK, Park S, Park JH, Jeong JS, Lee SH et al (2012) A comparative study of the transperitoneal and posterior retroperitoneal approaches for laparoscopic adrenalectomy for adrenal tumors. Ann Surg Oncol 19(8):2629–2634 22526902 10.1245/s10434-012-2352-0

[7] Assalia A, Gagner M (2004) Laparoscopic adrenalectomy. Br J Surg 91(10):1259–1274 15376201 10.1002/bjs.4738

[8] Lorenz K, Langer P, Niederle B, Alesina P, Holzer K, Nies C et al (2019) Surgical therapy of adrenal tumors: guidelines from the German association of endocrine surgeons (CAEK). Langenbecks Arch Surg 404(4):385–401 30937523 10.1007/s00423-019-01768-z

[9] Conzo G, Tartaglia E, Gambardella C, Esposito D, Sciascia V, Mauriello C et al (2016) Minimally invasive approach for adrenal lesions: systematic review of laparoscopic versus retroperitoneoscopic adrenalectomy and assessment of risk factors for complications. Int J Surg 28(Suppl 1):S118–S123 26708860 10.1016/j.ijsu.2015.12.042

[10] Hupe MC, Imkamp F, Merseburger AS (2017) Minimally invasive approaches to adrenal tumors: an up-to-date summary including patient position and Port placement of laparoscopic, retroperitoneoscopic, robot-assisted, and single-site adrenalectomy. Curr Opin Urol 27(1):56–61 27533502 10.1097/MOU.0000000000000339

[11] Groeben H, Nottebaum BJ, Alesina PF, Traut A, Neumann HP, Walz MK (2017) Perioperative alpha-receptor Blockade in Phaeochromocytoma surgery: an observational case series. Br J Anaesth 118(2):182–189 28100521 10.1093/bja/aew392

[12] Sherlock M, Scarsbrook A, Abbas A, Fraser S, Limumpornpetch P, Dineen R et al (2020) Adrenal Incidentaloma. Endocr Rev 41(6):775–820 32266384 10.1210/endrev/bnaa008 PMC7431180

[13] Brunt LM, Molmenti EP, Kerbl K, Soper NJ, Stone AM, Clayman RV (1993) Retroperitoneal endoscopic adrenalectomy: an experimental study. Surg Laparosc Endosc 3(4):300–306 8269248

[14] Walz MK, Peitgen K, Krause U, Eigler FW (1995) [Dorsal retroperitoneoscopic adrenalectomy–a new surgical technique]. Zentralbl Chir 120(1):53–58 7887040

[15] Walz MK, Giebler RM (1997) [Endoscopic, retroperitoneal adrenalectomy]. Chirurg 68(6):650 9324450

[16] Walz MK (2012) [Minimally invasive adrenal gland surgery. Transperitoneal or retroperitoneal approach?]. Chirurg 83(6):536–545 22653137 10.1007/s00104-011-2194-5

[17] Mihai R, De Crea C, Guerin C, Torresan F, Agcaoglu O, Simescu R, Walz MK (2024) Surgery for advanced adrenal malignant disease: recommendations based on European Society of Endocrine Surgeons consensus meeting. Br J Surg 111(1):znad266. 10.1093/bjs/znad266 10.1093/bjs/znad266 PMC10805373 38265812

[18] Alesina PF (2019) Retroperitoneal adrenalectomy-learning curve, practical tips and tricks, what limits its wider uptake. Gland Surg 8(Suppl 1):S36–S40 31404183 10.21037/gs.2019.03.11 PMC6646810

[19] Alesina PF, Knyazeva P, Hinrichs J, Walz MK (2022) Tailored approach in adrenal surgery: retroperitoneoscopic partial adrenalectomy. Front Endocrinol (Lausanne) 13:855326 35418944 10.3389/fendo.2022.855326 PMC8995530

[20] Conzo G, Pasquali D, Della Pietra C, Napolitano S, Esposito D, Iorio S et al (2013) Laparoscopic adrenal surgery: ten-year experience in a single institution. BMC Surg 13(Suppl 2):S5 24267584 10.1186/1471-2482-13-S2-S5 PMC3850966

[21] Kiernan CM, Lee JE (2019) Minimally invasive surgery for primary and metastatic adrenal malignancy. Surg Oncol Clin N Am 28(2):309–326 30851831 10.1016/j.soc.2018.11.011

[22] S2k-Leitlinie. Operative therapie von Nebennierentumoren. AWMF-Registernummer 088– 008. https://register.awmf.org/assets/guidelines/088-008l_S2k_Operative-Therapie_Nebennierentumoren_2019-07-abgelaufen.pdf

[23] Madani A, Lee JA (2019) Surgical approaches to the adrenal gland. Surg Clin North Am 99(4):773–791 31255206 10.1016/j.suc.2019.04.013

[24] Meng C, Du C, Peng L, Li J, Li J, Li Y et al (2021) Comparison of posterior retroperitoneoscopic adrenalectomy versus lateral transperitoneal laparoscopic adrenalectomy for adrenal tumors: A systematic review and Meta-Analysis. Front Oncol 11:667985 34041031 10.3389/fonc.2021.667985 PMC8142855

[25] Yip L, Duh QY, Wachtel H, Jimenez C, Sturgeon C, Lee C et al (2022) American association of endocrine surgeons guidelines for adrenalectomy: executive summary. JAMA Surg 157(10):870–877 35976622 10.1001/jamasurg.2022.3544 PMC9386598

[26] Feka J, Soliman B, Arikan M, Binter T, Hargitai L, Scheuba C et al (2024) Benefits of transitioning from transperitoneal laparoscopic to retroperitoneoscopic adrenalectomy-a single center experience. Gland Surg 13(11):1977–1985 39678401 10.21037/gs-24-286 PMC11635560

